# Measuring CO_2_ and ${\text{HCO}}_{3^{-}}$ permeabilities of isolated chloroplasts using a MIMS-^18^O approach

**DOI:** 10.1093/jxb/erx188

**Published:** 2017-06-19

**Authors:** Dimitri Tolleter, Vincent Chochois, Richard Poiré, G Dean Price, Murray R Badger

**Affiliations:** ARC Centre of Excellence for Translational Photosynthesis, Division of Plant Sciences, Research School of Biology, Australian National University, Canberra, ACT, Australia

**Keywords:** CCM, chloroplast bicarbonate permeability, chloroplast CO2 permeability, MIMS, photosynthesis

## Abstract

To support photosynthetic CO_2_ fixation by Rubisco, the chloroplast must be fed with inorganic carbon in the form of CO_2_ or bicarbonate. However, the mechanisms allowing the rapid passage of this gas and this charged molecule through the bounding membranes of the chloroplast envelope are not yet completely elucidated. We describe here a method allowing us to measure the permeability of these two molecules through the chloroplast envelope using a membrane inlet mass spectrometer and ^18^O-labelled inorganic carbon. We established that the internal stromal carbonic anhydrase activity is not limiting for this technique, and precisely measured the chloroplast surface area and permeability values for CO_2_ and bicarbonate. This was performed on chloroplasts from several plant species, with values ranging from 2.3 × 10^–4^ m s^–1^ to 8 × 10^–4^ m s^–1^ permeability for CO_2_ and 1 × 10^–8^ m s^–1^ for bicarbonate. We were able to apply our method to chloroplasts from an Arabidopsis aquaporin mutant, and this showed that CO_2_ permeability was reduced 50% in the mutant compared with the wild-type reference.

## Teaser

New approach to determine the inorganic carbon permeability across the chloroplast envelope, and to measure the role of aquaporin Pip1,2 on CO_2_ permeability.

## Introduction

The permeability of biological membranes to the gases CO_2_ and O_2_ is an extremely important property of cells of all microbial and multicellular organisms where respiration and photosynthesis are the most important energetic processes sustaining life. This is the reason why being able to measure the permeability of membranes through which these gases diffuse and to understand how sufficient permeability is achieved and modulated are of considerable interest (Endeward *et al.*, 2014; Kai and Kaldenhoff, 2014; Raven and Beardall, 2016). In this respect, this study is focused on measuring and understanding CO_2_ (and ${\text{HCO}}_{3^{-}}$) permeability of plant chloroplasts where photosynthesis is the major process occurring and CO_2_ is being consumed and O_2_ is evolved in equimolar amounts.

Our understanding of CO_2_ permeability has evolved over the past 100 years. Initially it was proposed that lipid-based biological membranes were very permeable to CO_2_, based on the lipid/water partitioning of the gas, and that they posed little diffusion resistance to its movement (Missner *et al.*, 2008; Endeward *et al.*, 2014). This view was supported by measurements of artificial lipid membrane systems (Missner *et al.*, 2008). However, over the past 10 years, it has became increasingly apparent that this diffusion can be greatly slowed by the inclusion of various components which are found in functional biological membranes such as sterols (cholesterol) and protein complexes, to create the possible reality that CO_2_ permeability may be restricted to an extent which requires the introduction of specialized CO_2_ transfer protein complexes to speed up CO_2_ entry. These transfer complexes are proposed to be members of the aquaporin family of water channel proteins found in most biological membranes (Boron *et al.*, 2011; Itel *et al.*, 2012; Endeward *et al.*, 2014; Kai and Kaldenhoff, 2014).

Our understanding of the CO_2_ permeability of biological membranes has been heavily dependent on the measurement techniques employed to study CO_2_ transfer in artificial lipid membranes and liposomes as well as intact biological cells and organelles (Endeward *et al.*, 2014). These methods have demonstrated that the various measurement techniques have intrinsic limitations and advantages with regard to their applicability and inherent capability to measure quantitatively accurate CO_2_ permeability values. Stopped flow spectrophotometry techniques tend to give comparatively low permeability values, being limited by the time resolution of the measurement techniques; mass spectrometric ^18^O exchange techniques give comparatively higher values, making use of the relatively slow kinetics of isotopic equilibrium and increased time resolution; and pH microelectrode techniques appear to give the highest values but are limited to use with relatively large surface area artificial membrane systems or large fixed position cells (Missner *et al.*, 2008). Based on these factors, we have focused on the use of MS ^18^O exchange techniques, which offer the ability to work with cells and chloroplasts containing carbonic anhydrase (CA) with a fast time resolution.

Our understanding of the transfer of CO_2_ into plant chloroplasts and its ultimate fixation by Rubisco into sugars depends on whether plants possess a CO_2_-concentrating mechanism (CCM) to enhance CO_2_ around Rubisco relative to the external ambient concentration. For C_3_ plants such a tobacco and wheat, it is assumed that they can be modelled as a simple passive diffusion of CO_2_ down a concentration gradient from the external air to the sites of Rubisco in the chloroplast. The diffusion pathway to CO_2_ is a series of resistances including the stomata, the cell wall and plasma membrane, the cytosolic pathway, and the chloroplast envelope. The non-stomatal resistances are responsible for determining the total mesophyll conductance, of which the chloroplast component is proposed to contribute ~30–40% (Tholen and Zhu, 2011). There is considerable interest in understanding what contributes to mesophyll conductance, and how it may differ, as variation in these resistances can contribute to photosynthetic efficiency when leaves are CO_2_ limited (Tholen *et al.*, 2012, 2014; Flexas *et al.*, 2013). For C_4_ plants such as maize and sorghum, the situation is quite different, with Rubisco being contained in the chloroplasts of the bundle sheath cells and CO_2_ being supplied by decarboxylation of a C_4_ acid. The primary passive supply of CO_2_ occurs in the cytosol of the mesophyll cells where CO_2_ is converted to ${\text{HCO}}_{3^{-}}$ by CA for primary fixation by phosphenolpyruvate (PEP) carboxylase (Hatch, 1987).

This study focuses on exploring how a mass spectrometric ^18^O exchange technique can be applied to isolated C_3_ plant chloroplasts to understand their CO_2_ and ${\text{HCO}}_{3^{-}}$ envelope conductance properties. The findings of the study demonstrate that the approaches taken with chloroplast isolation, membrane inlet mass spectrometry (MIMS), and theoretical modelling can be applied to obtain reasonable estimates of chloroplast CO_2_ permeability for a range of species, can also be extended to infer ${\text{HCO}}_{3^{-}}$ permeability values, and can demonstrate the differences in CO_2_ conductance caused by the presence of chloroplast membrane aquaporins.

## Materials and methods

### Plant material and growing conditions

Spinach (*Spinacea oleracae*) leaves were obtained fresh from local markets. *Arabidopsis thaliana* ecotype Col-0 and *atpip1;2-1* (Heckwolf *et al.* 2011) seeds, and *Nicotiana benthamiana* seeds were sown directly on to Debco seed raising potting mix (Debco Pty Ltd, Australia). After 2 weeks, 10 plants were transferred and grown in pots with a mix of Debco Plugger starter plus and Seed Raising (3:1 v:v) supplied with Scotts osmocote exact mini (1 g kg^–1^) (Scotts International, The Netherlands). *Pisum sativum* seeds were directly planted into a pot with the same potting mix. Plants were grown for ~45 d in a growth chamber under controlled conditions (16:8 h photoperiod, light at 250 µmol quanta m^–2^ s^–1^, 25 °C:20 °C day:night temperature, and relative humidity at 60%, watered every 2 d, for *N. benthamiana* and *P. sativa*); (8:16 h photoperiod, light at 250 µmol quanta m^–2^ s^–1^, 22 °C:22 °C day:night temperature and relative humidity at 60%, watered every 2 d, for *A. thaliana*).

Yeast (*Saccharomyces cerevisiae*) INV*S*c1: *MATa his3D1 leu2 trp1-289 ura3-52 MA*T *his3D1 leu2 trp1-289 ura3-52::human CA* were grown on YPD broth (–leucine,–uracil) at 29 °C overnight under continuous shaking.

### Chloroplast isolation

An 8–10 g aliquot of fresh leaves was ground for 2 s with a Polytron mechanical homogenizer (Kinematica Gmbh, Germany) in 20 ml of isolation buffer (sorbitol 330 mmol l^–1^, MOPS 30 mmol l^–1^ adjusted at pH 7.8, EDTA 2 mmol l^–1^, BSA 1.5 g l^–1^). After filtration through eight layers of miracloth (Wattman, USA), the extract was centrifuged at 1500 *g* for 90 s (Sorvall rotor SS34). The pellet was delicately resuspended in 4 ml of isolation buffer. Starch and nuclei were spun down by a centrifugation at 120 *g* for 45 s. Crude chloroplasts were concentrated to 500 μl by centrifugation (1500 *g*, 90 s) and then pipetted onto the top of a linear Percoll gradient (50% Percoll, sorbitol 330 mmol l^–1^, MOPS 30 mmol l^–1^ adjusted at pH 7.8, EDTA 2 mmol l^–1^, BSA 1.5 g l^–1^) previously autoformed by centrifugation for 1 h at 20 000 *g*. Pure chloroplasts were collected from the bottom fraction after centrifugation at 5000 *g* for 5 min and kept in the dark and on ice for <1 h before the permeability assay. Chloroplast integrity as assayed by reaction with ferricyanide (Mourioux and Douce, 1981) was found to be 80–85%. We routinely used phase-contrast microscopy (Leica DM5500 B, Germany) to monitor the integrity of the chloroplast preparation.

### Determination of size and number

Pure chloroplasts were examined under a fluorescence microscope (Leica DM5500 B, Germany) and then quantified using flow cytometry. Samples of diluted chloroplasts were analysed using Fortessa and LSRII cytometers (BD Biosciences, USA). Size was determined using forward scatter (FSC) intensity after calibration against size reference beads (1, 2, 4, 6, 10, and 15 µm; Flow cytometry size calibration kit, Molecular Probes, ThermoFisher Scientific, USA). Absolute chloroplast number was determined by mixing the sample with a calibrated suspension of microspheres that have specific fluorescence emissions (CountBright absolute counting beads; Molecular Probes, ThermoFisher Scientific). Data were processed using FlowJo software (Flow Jo LLC, USA) by plotting side scatter (SSC) against FSC or fluorescence against FSC. In parallel to this, the Chl *a* concentration was determined spectrophotometrically after pigment extraction in 100% methanol (MacKinney, 1941). Surface area was calculated for chloroplast particles, assuming they were spherical, and summed up to obtain the total surface value. This value was normalized by the absolute chloroplast count to give an average surface area per chloroplast and surface area injected per assay. The same has been done for the yeast suspension.

### Permeability assay

For low enrichment assays, 2.5 × 10^–3^ mol l^–1^ (final concentration) of low ^18^O-enriched NaH^13^CO_3_ (equilibrated against 1.2% [^18^O]water) was added to the reaction buffer (EPPS 100 mmol l^–1^ at pH 7.8, sorbitol 330 mmol l^–1^) in the mass inlet mass spectrometer (MIMS) cuvette (600 μl total volume, see Supplementary Fig. S1 at *JXB* online). Dextran-bound acetazolamide (Ramidus AB, Sweden) was also added (1.2 µg ml^–1^ to get an equivalent effect of 1.2 µmol ml^–1^ acetazolamide on bovine CA) to eliminate external CA activity. After chemical equilibration was reached (from 200 s to 300 s), chloroplast solution was added (<1/60th cuvette volume) in the dark, and the concentration of ^13^C^18^O^16^O (*m/z*=47) species was monitored over time by MIMS (Isoprime100, Isoprime, UK). In high enrichment assays, ^13^C^18^O^18^O (*m/z*=49) and ^13^C^16^O^16^O (*m/z*=45) were also monitored, and highly enriched [^18^O]bicarbonate for these assays was equilibrated against 99% [^18^O]water. ^18^O-enriched inorganic carbon (Ci) was prepared by incubating 0000.5 mol l^–1^ NaH^13^CO_3_ with either 1.2% (1% added labelled water plus 0.2% natural abundance in the unenriched water) or 99% H_2_^18^O in a sealed vial at room temperature for at least 24 h. In low enriched experiments, the added inorganic carbon was assumed to be in the form of ${\text{HCO}}_{3^{-}}$ with an enrichment of 3.5%, which was predicted from a model of labelling in 1.2% [^18^O]water. In-house Python scripts were used to record and process data from the mass spectrometer.

### Mathematical modelling procedures

**Fig. 1.**
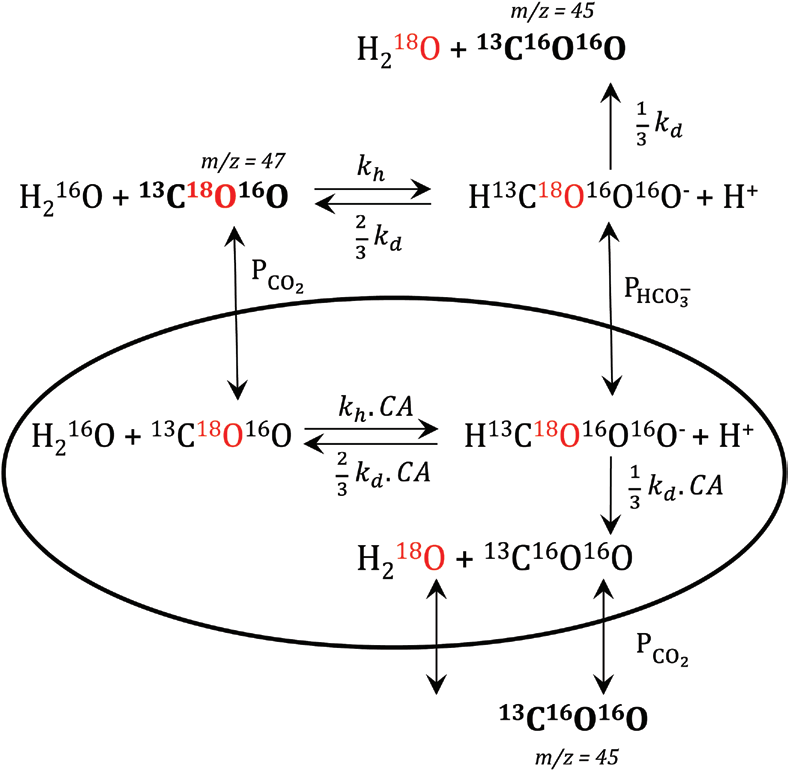
A diagram of the assumed exchange of inorganic carbon isotopes with water used in the mathematical simulations and modelling described herein. $P_{{\text{HCO}}_{3^{-}}}$, ${\text{HCO}}_{3^{-}}$ permeability of chloroplast; $P_{{\text{CO}}_{2}}$, CO_2_ permeability of chloroplast; *k*_h_, CO_2_ hydration rate constant; *k*_d_, ${\text{HCO}}_{3^{-}}$ dehydration rate constant; CA, scaling factor of carbonic anhydrase activity. As the loss of ^18^O from Ci species is the key component of measurements, ^18^O is labelled in red. Ci species measurable in the mass spectrometer are in bold, and mass over charge value (*m/z*) specified. ^13^C-labelled inorganic carbon is used for all assays.

Modelled time courses and curve-fitting of data shown herein were done with a biochemical network simulation program, COPASI, available on the internet (copasi.org) and described in detail by Hoops *et al.* (2006). We used COPASI to simulate time courses of reaction intermediate changes in a two-compartment model (external and chloroplast stroma) where reactants are linked in a biochemical network described in Fig. 1. We used the deterministic approach based on solving a set of differential equations (ODEs) shown in Supplementary Figs S3 and S4, and the modelling parameters described in Supplementary Tables S1 and S2.

We used the LSoda deterministic solving routine with a time step of 0.1 s and ran the model for 600 s. Initial concentrations of labelled CO_2_ and ${\text{HCO}}_{3^{-}}$ species were set as listed in Supplementary Table S1 and the injection of chloroplasts was simulated by changing the chloroplast envelope area from 0 to 30 m^2^ m^–3^ at 200 s or 300 s.

The COPASI software is able to fit the model to data from time course experiments. The curve fitting and parameter estimation is described by Hoops *et al.* (2006) and uses a weighted sum of squares minimization approach using the Levenberg–Marquardt algorithm provided by the program, and the root mean square was checked to be <5%. We used this capability to derive the actual experimentally observed hydration and dehydration rate constants in the assay medium (*k*_h_ and *k*_d_) from pre-experiments with highly enriched NaH^13^CO_3._ Those values are then used in the low enriched NaH^13^CO_3_ experiment with chloroplasts for the estimation of ${\text{P}}_{{\text{CO}}_{2}}$ and ${\text{P}}_{{\text{HCO}}_{{\text{3}}^{-}}}$ values for the assay.

## Results

### The ^18^O exchange technique

CO_2_ and ${\text{HCO}}_{3^{-}}$ permeability across biological membranes into an internal compartment containing CA can be studied using MIMS and ^18^O-labelled inorganic carbon. Techniques based on the kinetics of exchange of ^18^O from labelled Ci species with [^16^O]water have been used by two groups employing somewhat different approaches. Silverman and colleagues starting in 1974 (Silverman, 1974; Silverman *et al.*, 1976) studied CO_2_ and ${\text{HCO}}_{3^{-}}$ permeability in red blood cells using highly ^18^O-enriched inorganic carbon (equilibrated with highly ^18^O-enriched water). They demonstrated permeabilities to both CO_2_ and bicarbonate and bicarbonate exchange which were facilitated by Cl^–^/${\text{HCO}}_{3^{-}}$ exchange activity of the band III protein anion exchanger. They developed mathematical analysis techniques to derive CO_2_ and ${\text{HCO}}_{3^{-}}$ permeability values from time courses, which followed the changes in CO_2_ isotopes after the sequential addition of labelled Ci and then erythrocytes over a period of ≥10 min.

Subsequently, Itada and Forster (1977) explored the application of a similar technique, using low enriched [^18^O]Ci species (equilibrated with 1.2% ^18^O-enriched water, see the Materials and methods) to develop a simplified mathematical approach (as fewer labelled species are involved) to study the same phenomena (Itada and Forster, 1977). This approach has been subsequently developed further using numerical curve-fitting procedures to describe the operation and limitations of this system in greater detail (Wunder and Gros, 1998; Wunder *et al.*, 1998). We have used a similar approach with a reduced enrichment procedure to determine CO_2_ and ${\text{HCO}}_{3^{-}}$ permeability of isolated chloroplasts, and have tested some hypotheses with highly ^18^O-enriched Ci (details of our procedures are described in the Materials and methods). Figure 1 shows the chemical and diffusion reactions, which we have assumed to occur when creating the mathematical model which we have used to simulate and analyse the results obtained from our time course experiments shown in Fig. 6.

**Fig. 2.**
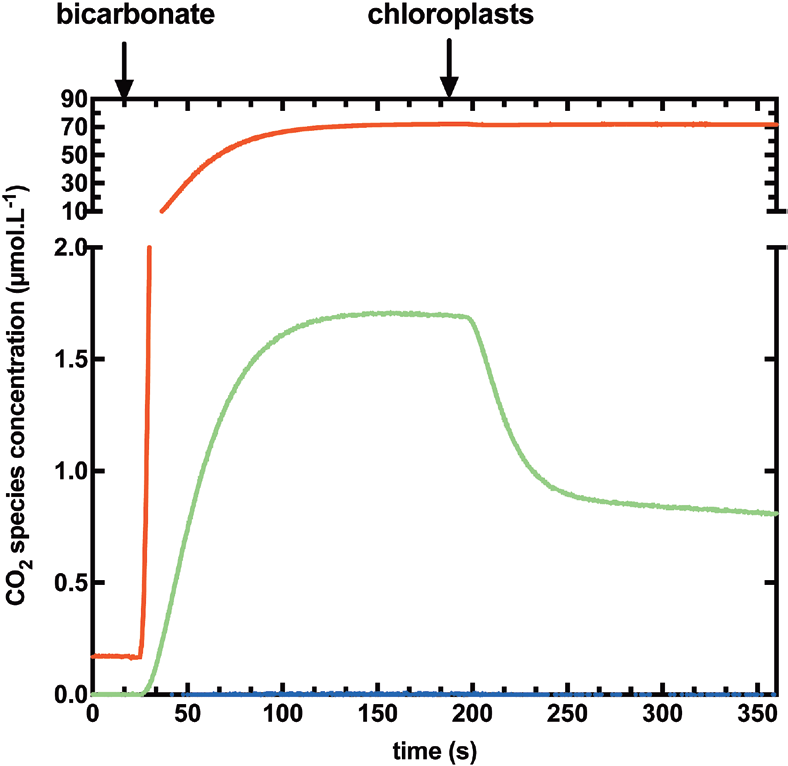
Typical time course for a low ^18^O-enriched permeability assay. Changes in the concentrations of ^13^C^18^O^18^O (*m/z*=49, blue), ^13^C^18^O^16^O (*m/z*=47, green), and ^13^C^16^O^16^O (*m/z*=45, red) species are shown. After injection of low ^18^O-enriched Ci (equilibrated against 1.2% [^18^O]water), chemical equilibrium is reached before chloroplast injection at 200 s.

**Fig. 3.**
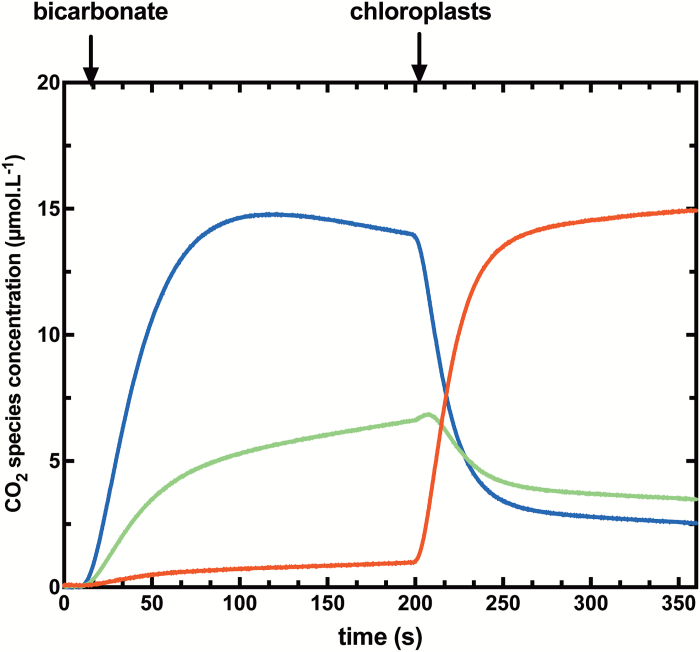
Typical time course for a highly ^18^O-enriched assay. Changes in concentrations of ^13^C^18^O^18^O (*m/z*=49, blue), ^13^C^18^O^16^O (*m/z*=47, green), and ^13^C^16^O^16^O (*m/z*=45, red) species are shown. Conditions were the same as Fig. 2 but using highly enriched [^18^O]Ci equilibrated against 99% [^18^O]water, instead of low ^18^O-enriched Ci.

We have produced time courses for experiments conducted with chloroplasts using both low (Fig. 2) and highly (Fig. 3) enriched Ci species. After reaching a chemical equilibrium between CO_2_ and ${\text{HCO}}_{3^{-}}$, chloroplasts are added in the dark, initiating a rapid drop of ^18^O-labelled CO_2_ species (*m/z*=49 and *m/z*=47), followed by a new isotopic equilibrium. Permeabilities are derived from the study of these kinetics. The primary difference between the two experimental systems is that in the highly enriched system, all three masses of labelled CO_2_ isotopes (*m/z*=49, m/z=47, and *m/z*=45) are significantly present and are changing, whereas singly labelled CO_2_ (*m/z*=47) is the dominant ^18^O-labelled CO_2_ species which changes over time in the low enriched system. In this condition, ^13^C^18^O^16^O (*m/z*=47) represent ~1–2% of total CO_2_ species in the solution (Fig. 2).

### Assumption about internal CA activity

One important assumption of this technique and modelling is that there is sufficient CA within the internal compartment (stroma of the chloroplast) to equilibrate the ^18^O isotope rapidly between the Ci species and the water. Thus, it is important to test this assumption. In this regard, although the singly labelled species experiment is simpler to analyse, it conveys less information on the extent of the isotopic equilibrium catalysed by CA within the chloroplasts due to the lack of an intermediate CO_2_ species (*m/z*=47 in Fig. 3), which can convey information about incomplete isotopic exchange.

As it is not possible to measure the stromal (internal) CA activity of our chloroplast preparations, we simulated the isotopic exchange between labelled Ci species and water (in a scenario of highly enriched [^18^O]bicarbonate) assuming a range of CA activities (10^3^- to 10^6^-fold increased exchange rates compared with the uncatalysed reaction) (Supplementary Fig. S2). A ≥10^6^-fold increase in exchange rate is necessary to reach complete exchange with stromal water. Below this activity, production of singly ^18^O-labelled CO_2_ (*m/z*=47) occurs after the chloroplast injection. In our experience, we have observed consumption of singly ^18^O-labelled CO_2_ (*m/z*=47) when we inject the chloroplasts (Fig. 3), indicating that CA activity inside the chloroplast appears sufficient to achieve at least a 10^6^-fold increase in Ci/water exchange rates. It should be noted that there are differences in the overall kinetics of the modelling and experimental results. In particular, mass 47 (singly labelled CO_2_) continues to rise in the modelling after the initial drop caused by injection of chloroplasts, and this does not happen in the actual experiments with chloroplasts (Fig. 3; Supplementary Fig S7). This may indicate a deficiency in the modelling.

### Measuring chloroplast dimensions

A key parameter of our mathematical model and all permeability calculations is A, the area of chloroplast envelope per volume of assay (Supplementary Fig. S3; Supplementary Table S1). Surprisingly, the size and shape of isolated chloroplasts are not well described in the literature. An estimation of size and number on a small subpopulation by microscopy gives low precision data (for a review on cell counting, see Guillard and Sieracki, 2005). Indeed haemocytometer counts have low precision (within ±20% of the true count) due to random error and the relatively small numbers of cells counted, even when many replicate counts are made (Pringle and Mor, 1975; reviewed by Guillard *et al.*, 2005). In addition, counts are frequently inaccurate due to systematic errors (Berkson *et al.*, 1940; Pringle and Mor, 1975; Guillard and Sieracki, 2005).

**Fig. 4.**
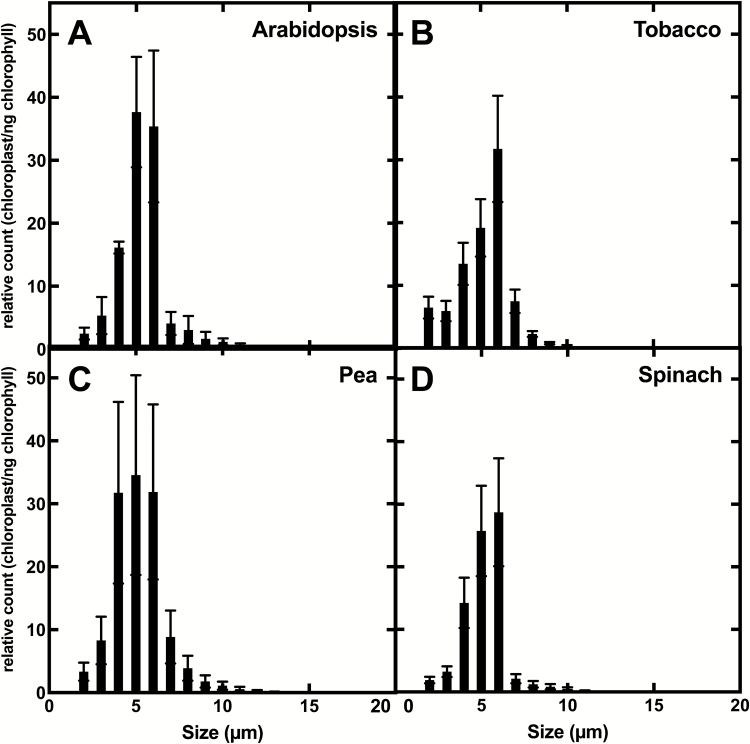
Chloroplasts size distribution for four C_3_ plant species, Arabidopsis (*Arabidopsis thaliana)* (A), tobacco (*Nicotiana benthamiana)* (B), pea (*Pisum sativa)* (C), and spinach (*Spinacea oleracea*) (D). Size and number were determined by flow cytometry, and are normalized by Chl *a* concentration.

We chose to use flow cytometry to determine the diameter and the number of chloroplasts in our samples because a large number of individual chloroplasts can be counted, providing a high intrinsic precision to the counts. In addition, the technique describes the diversity of size and shapes present in the sample. Raw data from flow cytometry (the ratio between SSC and FSC) did not show a strong ellipsoid shape of the chloroplasts (data not shown), and consequently we assumed chloroplasts to be spherical for surface area calculations. Regardless of species origin, the majority of purified chloroplasts we obtained have a diameter between 4 µm and 6 µm (Fig. 4). As explained in the Materials and methods, we are calculating the surface area of each chloroplast for ~100 000–200 000 counts, and obtain the absolute concentration of chloroplasts and their surface area in our preparations. Surface area per chloroplast is given in Table 1.

**Table 1.** Determination of chloroplast permeabilities (CO_2_ and ${\text{HCO}}_{3^{-}}$) for different plant species as described in Fig. 6; shown are means ± SDs (*n*=3 different chloroplast isolation from three different plants) Also included are values for yeast expressing human CA internally (from three independent cultures). Average surface area for each chloroplast or yeast was determined by flux cytometry

	$P_{{\text{CO}}_{2}}$ (m s^–1^)	${\text{HCO}}_{3^{-}}$ (m s^–1^)	Surface (m^2^) per unit
Plant chloroplast			
Spinach	6.9 × 10^–4^±1.24 × 10^–4^	9.9 × 10^–9^±5 × 10^–10^	1.27 × 10^–10^±2.78 × 10^–11^
Tobacco	3.9 × 10^–4^±2.0 × 10^–5^	1.1 × 10^–8^±4 × 10^–10^	1.21 × 10^–10^±4.92 × 10^–11^
Pea	8.0 × 10^–4^±5.5 × 10^–5^	9.8 × 10^–9^±9 × 10^–10^	1.22 × 10^–10^±3.79 × 10^–11^
Arabidopsis	2.3 × 10^–4^±6.4 × 10^–5^	1.0 × 10^–8^±5 × 10^–10^	1.24 × 10^–10^±4.68 × 10^–11^
Yeast (*S. cerevisiae*)			
INV*S*c1: *MATa his3D1 leu2 trp1-289 ura3-52 MA*T *his3D1 leu2 trp1-289 ura3- 52::human CA*	1.09 × 10^–3^±1.72 × 10^–4^	9.7 × 10^–9^±1 × 10^–10^	2.56 × 10^–10^±5.75 × 10^–11^

### Modelling and measuring CO_2_ and ${\text{HCO}}_{3^{-}}$ permeabilities

**Fig. 5.**
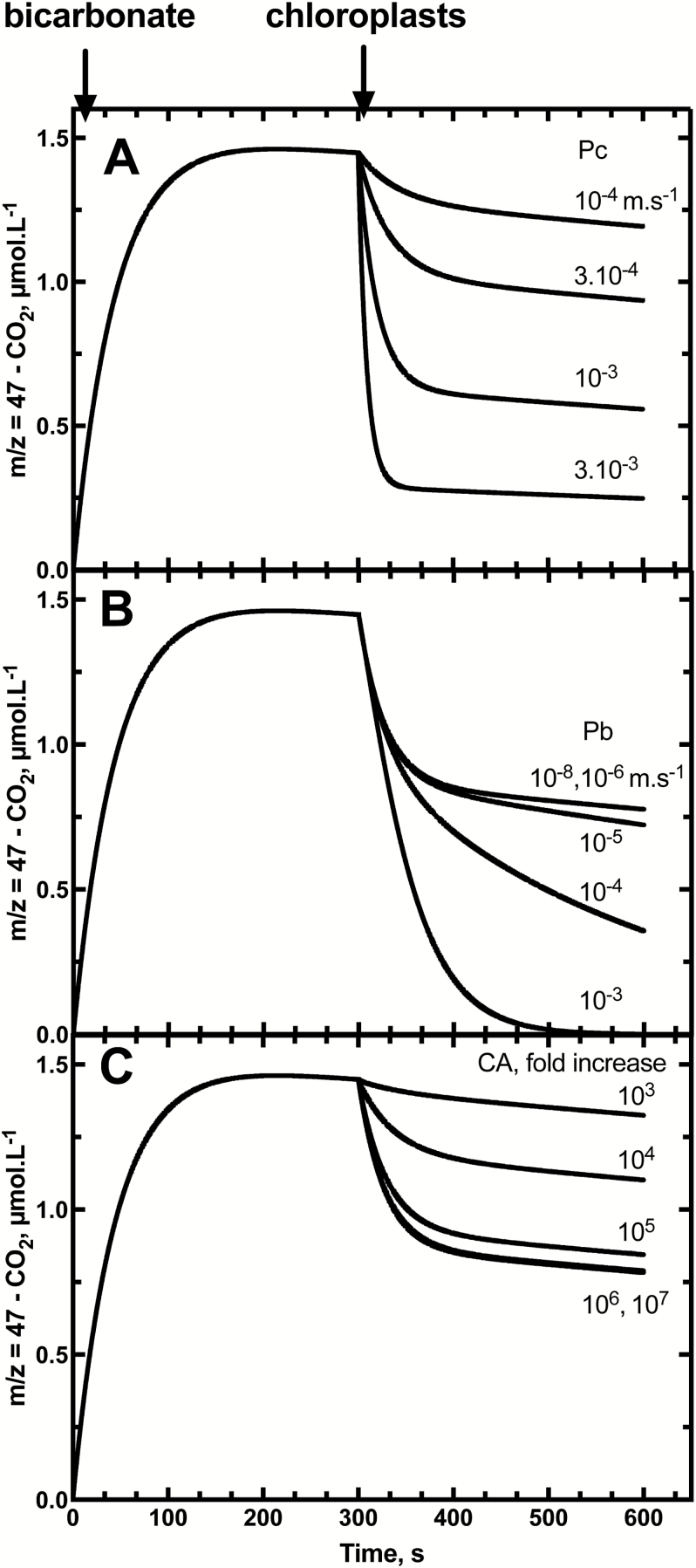
The modelled effects of variation in chloroplast envelope CO_2_ permeability ($P_{{\text{CO}}_{2}}$; A), ${\text{HCO}}_{3^{-}}$ permeability (${\text{HCO}}_{3^{-}}$; B), and in internal chloroplast CA activity (C) on the decrease in ^13^C^18^O^16^O (*m/z*=47) species in the assay after the injection of chloroplasts. In (A) $P_{{\text{CO}}_{2}}$was varied from 10^–4^ m s^–1^ to 3 × 10^–3^ m s^–1^ ($P_{{\text{CO}}_{2}}$was fixed at 10^–8^ m s^–1^, CA at 10^7^-fold increase). In (B) $P_{{\text{HCO}}_{3^{-}}}$was varied from 10^–8^ m s^–1^ to 10^–3^ m s^–1^ ($P_{{\text{CO}}_{2}}$ was fixed at 5 × 10^–4^ m s^–1^, CA at 10^7^-fold increase). In (C), CA activity was modelled as the fold increase in the interconversion between CO_2_ and ${\text{HCO}}_{3^{-}}$ within the chloroplast stroma and was varied between a 10^3^ and 10^7^ increase in the hydration and dehydration rate constants ($P_{{\text{CO}}_{2}}$was fixed at 5 × 10^–4^ m s^–1^ and $P_{{\text{HCO}}_{3^{-}}}$ at 10^–8^ m s^–1^). The modelling procedures are described in the Materials and methods and supplementary information, based on the use of low ^18^O-enriched bicarbonate equilibrated against 1.2% [^18^O]water. Labelled bicarbonate is added at time zero (1 mmol l^–1^ total) and chloroplasts are added after 300 s of equilibration in the assay.

Isotopic exchange between labelled Ci (in the scenario of low ^18^O-enriched bicarbonate equilibrated against 1% [^18^O]water) and water was mathematically simulated to predict the effect of variation of CO_2_ and ${\text{HCO}}_{3^{-}}$ permeability (Fig. 5A, B), and chloroplast CA activity (Fig. 5C). This modelling used chloroplast dimensions per assay similar to those observed in actual assays (Tables 1, 2; Supplementary Table S2). After chemical equilibration between CO_2_ and bicarbonate during the first 300 s, the addition of chloroplasts induced an obvious consumption of labelled CO_2_ (*m/z*=47). For CO_2_ permeabilities ranging from 3 × 10^–3^ m s^–1^ to 10^–4^ m s^–1^, this drop is measurable and significantly different from one condition to the other (Fig. 5A). Significant differences are also present if the bicarbonate permeability varies between 10^–6^ m s^–1^ and 10^–3^ m s^–1^ (Fig. 5B).

Similar to what is shown in Supplementary Fig. S2, internal CA activity has an influence on the kinetics of isotopic exchange. If the CA activity increases the CO_2_ hydration rate and bicarbonate dehydration rate by 1000-fold, the drop of labelled CO_2_ (*m/z*=47) is too small to be measured (Fig. 5C). With 10^6^- and 10^7^-fold increases in the catalysis rate, the drop and time course is similar, and has reached its maximum indicating, as in Supplementary Fig. S2, that a 10^6^-fold increase is sufficient to achieve complete isotopic exchange of internal Ci species with water.

**Fig. 6.**
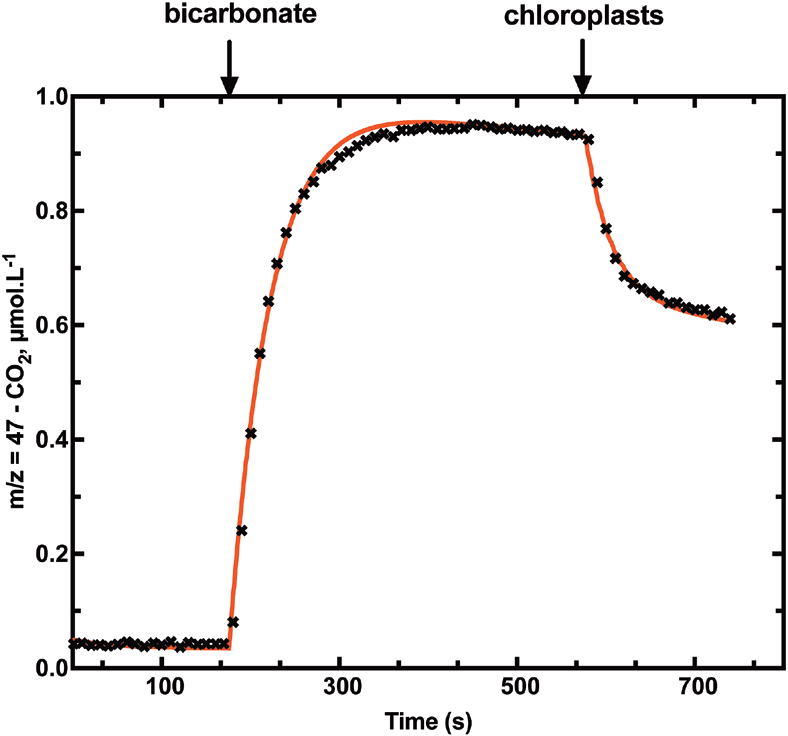
Time course (black crosses) of ^13^C^18^O^16^O (*m/z*=47) and curve fitting (red line) using COPASI software as described in the Materials and methods. Low ^18^O-enriched Ci (2.5 mM) was injected at *t*=170 s, and chloroplasts at *t*=577 s after chemical equilibrium had been reached.

The curve fitting of our experimental time courses has been performed with a biochemical network simulation program, COPASI (Fig. 6), using the ODEs presented in Supplementary Fig. S3. This approach was used to obtain the values of CO_2_ permeability and bicarbonate permeability reported in Table 1. However, it has also been possible to estimate CO_2_ permeability graphically from chloroplast injection time courses as shown in Supplementary Fig. S4. Values obtained with this empirical method have been compared with values assumed in the modelled time course shown in Fig. 5A, and show a very strong linear correlation (Supplementary Fig. S6). We used this technique to determine ${\text{P}}_{{\text{CO}}_{2}}$ values rapidly for a number of experiments. Supplementary Fig. S7 shows that our assay methods appear valid for a wide range of chlorophyll concentrations per assay (0.2–3 µg Chl ml^–1^) but there is a curvilinear relationship when injection volumes are increased (Supplementary Fig. S8) and this is evident above 10 µl per 600 µl assay. Our assay procedures used <10 µl of chloroplast solution per assay.

### Measurements from a range of species

We have performed CO_2_ and bicarbonate permeability assays on chloroplasts from a range of different C_3_ plants. As described previously, we have determined precisely the surface of the interface between the outside and the inside (stroma and thylakoids of chloroplast). With our isolation conditions, all isolated chloroplasts have a similar size for the different species tested (Fig. 4) and so surface areas per assay are comparable in our experiments. Where bicarbonate permeability is nearly identical for all species at 10^–8^ m s^–1^, CO_2_ permeability varies from 2.3 × 10^–4^ m s^–1^ for Arabidopsis to 8.0 × 10^–4^ m s^–1^ for pea.

For comparison, we have also tested our method on yeast cells overexpressing human CA internally. We have checked that CA activity was sufficient to carry out our method (Supplementary Fig. S9), and the time course with the highly enriched Ci (in ^18^O) is similar to the chloroplasts, with an activity estimated as a >10^6^-fold increase. In yeast, bicarbonate permeability was low, similar to chloroplasts, but CO_2_ permeability is higher at 1 × 10^–3^ m s^–1^.

### Arabidopsis aquaporin knockouts

**Fig. 7.**
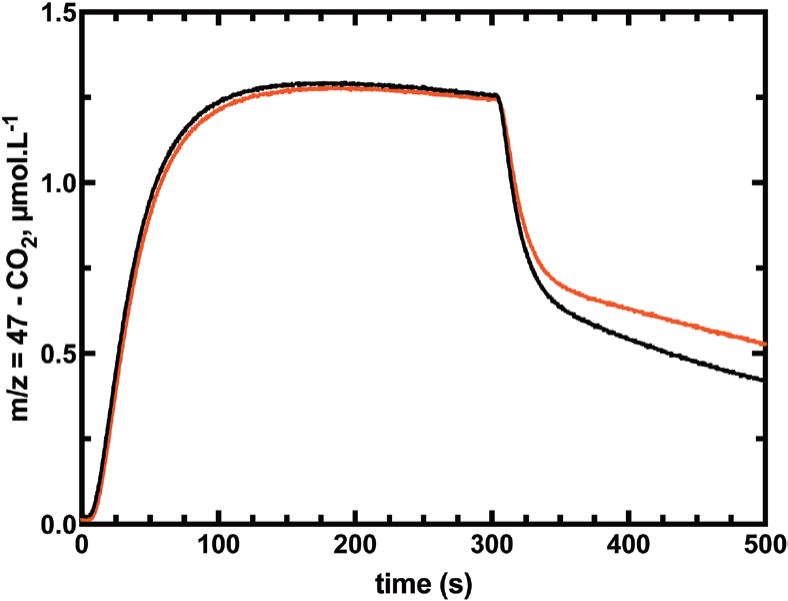
Time course of ^13^C^18^O^16^O (*m/z*=47) species for the wild type and PIP1.2 mutants. Low ^18^O-enriched Ci (2.5 mmol l^–1^) was injected at *t*=5 s, and chloroplasts from the wild type (black) or PIP1,2 (red) were injected at *t*=300s after chemical equilibrium had been reached. Final chlorophyll concentrations were 1.9 μg ml^–1^ and 2.0 μg ml^–1^ for the wild type and mutant, respectively.

In order to test our method on estimating CO_2_ permeability, we chose an Arabidopsis mutant that has been described to have a lower CO_2_ permeability, *atpip1;2-1* (Heckwolf *et al.*, 2011). This is a knockout mutant of an aquaporin, orthologous to NtAQP1 which is proposed to facilitate CO_2_ diffusion across membranes (Uehlein *et al.*, 2008). Despite the lack of absolute evidence of the localization of NtAQP1, different proteomic studies have found it in the chloroplast envelope (Beebo *et al.*, 2013). In our experiments, the decrease in ^13^C^18^O^16^O (*m/z*=47) species in the assay after the injection of chloroplasts is lower for the mutant *atpip1;2-1* than for the wild type (Fig. 7). This difference results in a calculated CO_2_ permeability of *atpip1;2-1* which is half that of the wild type at 10^–4^ m s^–1^ (Table 2).

**Table 2.** Measurements of chloroplast permeability for the *Arabidopsis thaliana* wild type and *atpip1;2-2* mutant Chloroplast permeabilities were determined using the same principle as described for Table 1; shown are means ± SDs (*n*=4 different chloroplast isolations from four different plants)

Chloroplast permeability	Wild type	*atpip1;2-1*
${\text{P}}_{{\text{CO}}_{2}}$ (m s^–1^)	2.27 × 10^–4^±6.6 × 10^–5^	1.02 × 10^–4^±5.9 × 10^–5^
${\text{P}}_{{\text{HCO}}_{{\text{3}}^{-}}}$ (m s^–1^)	1.0 × 10^–8^±5 × 10^–10^	1.0 × 10^–8^±7 × 10^–10^

## Discussion

This study demonstrates that MIMS-^18^O Ci exchange techniques can be used to measure the CO_2_ and ${\text{HCO}}_{3^{-}}$ permeabilities of isolated chloroplasts. For simple measurements of permeability, it is most convenient to use low ^18^O-enriched Ci species where changes in singly labelled CO_2_ (*m/z*=47) are followed, as it allows simpler mathematics for curve fitting and parameter estimation. However, the use of highly ^18^O-enriched Ci species can be complementary when establishing the validity of assumptions concerning internal CA activity. The measurements with isolated chloroplasts from a range of species give ${\text{P}}_{{\text{CO}}_{2}}$ values that are close to those estimated to be necessary to support measured CO_2_ gas exchange within the chloroplast in the C_3_ leaf supported by passive CO_2_ diffusion (Evans *et al.*, 2009; Tholen and Zhu, 2011). However, a range of values was obtained from different species (8 × 10^–4^–2.3 × 10^–4^ m s^–1^) which indicates either real variation between species or other variable limitations of the technique which are discussed below. In addition to measuring CO_2_ permeability, the technique also established that all chloroplasts had a consistently low permeability to ${\text{HCO}}_{3^{-}}$ (10^–8^ m s^–1^) which has never been directly measured before, although this has been inferred from previous studies of Ci accumulation within the chloroplast following the pH gradient between the inside and outside (Werdan and Heldt, 1972)

Our results with yeast allow us to compare our techniques with others in the literature. It is possible to find different values for CO_2_ permeability ranging from 5 × 10^–5^ m s^–1^ (Heckwolf *et al.*, 2011) to 1.7 × 10^–4^ m s^–1^ (Navarro-Ródenas *et al.*, 2012), passing through 10^–4^ m s^–1^ (Ding *et al.*, 2013). Those three studies employ the method of stopped flow based on intracellular pH changes caused by CO_2_ injection. In this study using the MIMS-^18^O approach, we are measuring a ${\text{P}}_{{\text{CO}}_{2}}$ of ~10^–3^ m s^–1^ which is 10 times higher than in these previous studies. The particular characteristics and limitations of the various measurement techniques have been previously reviewed (Endeward *et al.*, 2014), and limitations with time resolution reduce the CO_2_ permeabilities derived from the stopped flow technique. The MIMS-^18^O technique has fewer limitations with time resolution but there are other inherent sources of error, which need to be considered, and these are discussed below.

Our model to calculate CO_2_ and ${\text{HCO}}_{3^{-}}$ permeabilities of isolated chloroplasts is based on a number of important assumptions. A major assumption concerns the chloroplast CA activity, which needs to be sufficient for complete isotopic equilibration of Ci species and water within the chloroplast. To validate this hypothesis, we used highly enriched [^18^O]bicarbonate in our experiments and modelling of the impact of CA activity variation on predicted assay time courses. Modelling shows that a >10^5^-fold increase in the Ci–water interconversion rate over the uncatalysed rate is needed to achieve this both with low and highly enriched Ci techniques (Figs 2, 3; Supplementary Fig. S2). Experiments with tobacco chloroplasts indicate that there is sufficient CA within an isolated C_3_ chloroplast to achieve this (Fig. 3) and is consistent with calculations which indicate in tobacco that CA activity in the chloroplast is sufficient to speed up interconversion by >4 × 10^5^-fold (Price *et al.*, 1994). If, however, stromal CA was to be limiting, Fig. 5 shows that this would result in underestimating the true ${\text{P}}_{{\text{CO}}_{2}}$ value. There have been examples where the *in vivo* limiting CA activity level has been estimated by model fitting procedures and corrected ${\text{P}}_{{\text{CO}}_{2}}$ values obtained (Endeward and Gros, 2005). However, if CA activity levels are in fact significantly limiting, then this approach is likely to introduce its own errors. We have avoided doing this and prefer to verify the existence of sufficient CA levels where this correction does not need to be made.

Another important assumption is that we can precisely determine chloroplast surface area separating the external compartment from the stroma, as this is the interface at which transport and diffusion of CO_2_ and ${\text{HCO}}_{3^{-}}$ occur and is a significant calculation input. To achieve this, we chose a flow cytometry approach to obtain an average chloroplast size measurement on a large sampling of the isolated chloroplast preparation. However, in addition to chloroplast counts, it is also important to approximate the geometric shape of the chloroplasts for surface area calculations. This approximation can introduce a bias in our permeability value. In our set of data, the choice of a spherical model instead of an elliptical model would decrease the permeability value by ~4%. However, we chose the spherical model with regard to the images from optical microscopy and indications from the ratio (~1) between the side scatter and the forward scatter in flow cytometry.

In reality, a leaf is composed of different cell layers and types, which contain chloroplasts of different shape and/or size. As measurements shown here are derived from chloroplasts isolated using a particular technique, this may introduce biases. Chloroplasts are being isolated from all leaf cell types, so we have a heterogeneous population of chloroplasts in our preparation as shown in the size distribution (Fig. 4). Our permeability values are an average of all the chloroplasts in the cuvette so there will be an attenuation of any differences in chloroplast permeability between different tissues. In addition, the isolation procedure itself could favour the isolation of a subpopulation of chloroplasts which are more resistant to the isolation protocol, and so obtain the permeability of one specific class of chloroplast. Differences in ${\text{P}}_{{\text{CO}}_{2}}$ between the species in Table 1 may in part be due to some of the isolation biases and their differences between species.

We have ignored the influence of unstirred layers both inside and outside the chloroplast which have been identified as factors influencing the MIMS assay (Endeward and Gros, 2009), which have the potential to lead to underestimation of permeabilities of red blood cells by ~30%. However, we have considered their influence to be minor in our assays. In addition, we ignored any influence of H_2_^18^O accumulation within the chloroplast and the presence of 0.2% natural abundance in the reaction water. We have calculated the latter assumption to reduce P_CO2_ values by ~20% (data not shown).

Given the observed time courses of the permeability assays and the predicted modelling, the MIMS-^18^O technique shows good sensitivity for the measurement of ${\text{P}}_{{\text{CO}}_{2}}$ values. Given the dimensions of chloroplasts and chlorophyll concentrations of 0.2–3 μg Chl ml^–1^, we roughly estimate that the technique provides the ability to resolve ${\text{P}}_{{\text{CO}}_{2}}$ values in the range of 10^–2^–10^–5^ m s^–1^, which is well within the calculated values for the isolated chloroplasts. The situation for resolving potential variation in ${\text{HCO}}_{3^{-}}$ permeabilities is perhaps more limited. The technique certainly establishes that the chloroplast is relatively impermeable to ${\text{HCO}}_{3^{-}}$, but modelling would appear to show that it is difficult to resolve differences for ${\text{P}}_{{\text{HCO}}_{{\text{3}}^{-}}}$ below 10^–6^ m s^–1^.

The measurement technique used here, as is also the case for other methods, obviously requires that assays be conducted in the dark, as uptake of CO_2_ species by Rubisco in the light would make the technique unworkable. This raises the question of whether the envelope interface properties of the chloroplasts are affected by light. For example, if there was light activation of a CO_2_-conducting aquaporin then this would not be easily detected and could lead to the underestimation of ${\text{P}}_{{\text{CO}}_{2}}$ values. Similar arguments could be made for a light-stimulated pathway for ${\text{HCO}}_{3^{-}}$ entry if this existed.

Despite these potential errors, the observed ${\text{P}}_{{\text{CO}}_{2}}$ values are approaching the range which has been predicted from C_3_ leaf gas exchange measurements and modelling to be necessary to explain photosynthetic CO_2_ flux rates based on passive CO_2_ diffusion assumptions and models (Evans *et al.*, 2009; Tholen and Zhu, 2011). Previous measurements of ${\text{P}}_{{\text{CO}}_{2}}$ values for C_3_ chloroplasts using stopped flow techniques have reported values which are 1–2 orders of magnitude lower than those necessary for photosynthetic CO_2_ fluxes (Uehlein *et al.*, 2008). This indicates that the MIMS-^18^O technique has the ability to reveal chloroplast permeability values which are more realistic despite the limitations discussed above. In this context, we can readily detect differences in ${\text{P}}_{{\text{CO}}_{2}}$ between Arabidopsis chloroplasts isolated from the wild type and *atpip1,2* mutants (Table 2).

## Supplementary data

Supplementary data are available at *JXB* online.

Fig. S1. The MIMS assay cuvette design.

Fig. S2. The modelled effects of variation in internal chloroplast carbonic anhydrase activity on assays using highly enriched [^18^O]bicarbonate equilibrated against 99% [^18^O]water.

Fig. S3. Differential equations used for numerical modelling of time courses for changes in singly labelled CO_2_ species.

Fig. S4. Differential equations used for numerical modelling of time courses for changes in highly enriched [^18^O]bicarbonate equilibrated against 99% [^18^O]water in Fig. S2.

Fig. S5. Estimation of ${\text{P}}_{{\text{CO}}_{2}}$ from chloroplast injection time courses as shown in Fig. 2.

Fig. S6. Empirical verification of the graphical estimation procedure for the method and equation shown in Fig. S3.

Fig. S7. Correlation between chloroplast number (chlorophyll concentration) and determination of permeability values before correction by chloroplast number.

Fig. S8. Correlation between volume of chloroplasts injected in the MIMS cuvette and the drop in ^13^C^18^O^16^O (*m/z*=47) at the injection.

Fig. S9. Typical time course for a highly ^18^O-enriched assay with yeast injection.

Table S1. Parameters and their units and values for the model equations used in Fig. S3.

Table S2. Parameters and their units and values for the model equations used in Fig. S4.

## Data deposition

COPASI files of numerical modelling used for the generation of time courses for changes in labeled CO_2_ species. Dryad Digital Repository. http://dx.doi:10.5061/dryad.2r05d.

## Supplementary Material

Supplementary_Figures_S1-S9_tables_S1-S2

## Abbreviations:

CA: carbonic anhydrase
CCM: CO_2_-concentrating mechanism
Chl: chlorophyll
Ci: inorganic carbon
FSC: forward scatter
MIMS: membrane inlet mass spectrometry
SSC: side scatter.
