# Supplementary material for: Measuring CO2 and HCO3− permeabilities of isolated chloroplasts using a MIMS-18O approach
Source: J Exp Bot. 2017 Jun 19;68(14):3915–24. doi: 10.1093/jxb/erx188 (PMC5853524; doi:10.1093/jxb/erx188)
Supplement: Supplementary_Figures_S1-S9_tables_S1-S2 [file erx188_suppl_supplementary_figures_s1-s9_tables_s1-s2.pdf]

Supplementary data

Measuring CO_2 and HCO_3^- permeabilities of isolated chloroplasts using a MIMS-^{18}O approach

Dimitri Tolleter, Vincent Chochois, Richard Poiré, G. Dean Price and Murray R Badger

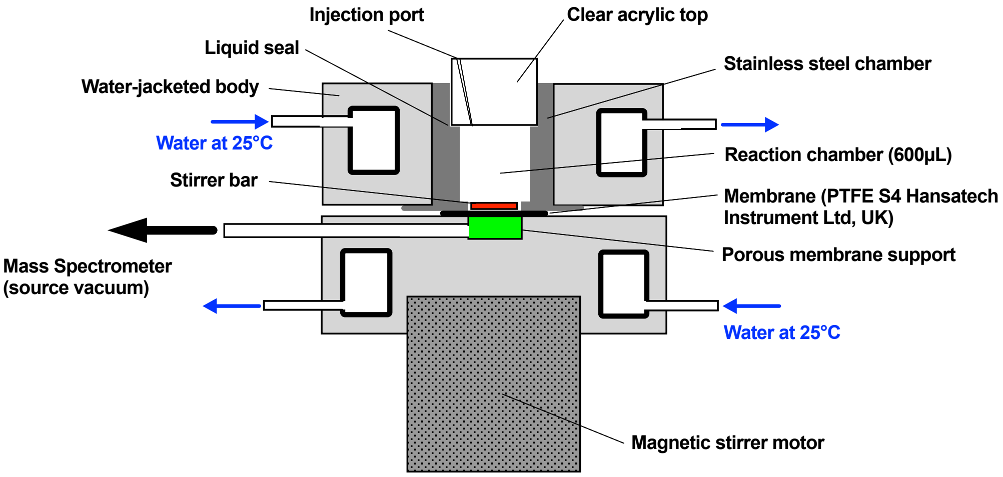

Figure S1
The MIMS assay cuvette design. In-house design of our 600µL cuvette (6.9mm diameter for 17 mm high)
To minimise boundary layers in liquid phase measurements a magnetic stirrer at 1500rpm is placed directly on the semi-permeable membrane.

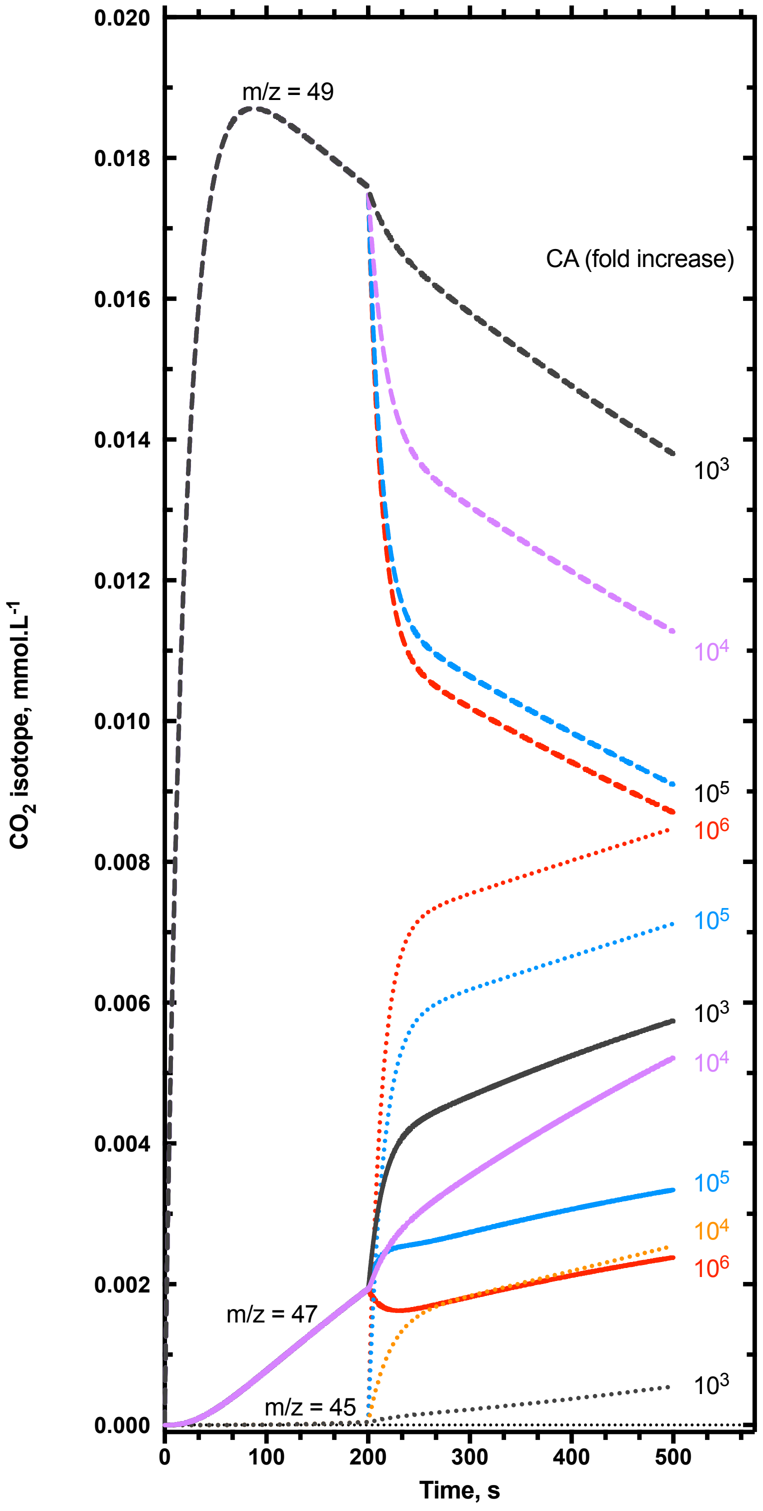

Figure S2
The modelled effects of variation in internal chloroplast carbonic anhydrase activity on assays using highly enriched ^{18}O bicarbonate equilibrated against 99% ^{18}O water. CA activity was modelled as the fold increase in the interconversion between CO_2 and HCO_3^- within the chloroplast stroma and was varied between a 10^3 and 10^6 (10^3 in black, 10^4 in purple, 10^5 in blue, 10^6 in red) increase in the hydration and dehydration rate constants. P_{CO_2} was 10^{-3} m s^{-1} and $P_{HCO_3^-}$ 10^{-8} m s^{-1}. Unlabelled ($^{13}C^{16}O_2$ m/z = 45; dotted line) singly labelled ($^{13}C^{18}O^{16}O$ m/z = 47; solid line) and doubly labelled CO_2 ($^{13}C^{18}O_2$ m/z = 49; dashed line) species are shown. The modelling procedures are described in the methods. Labelled bicarbonate is added at time zero (1 mmol L^{-1} total) and chloroplasts are added after 200 s of equilibration in the assay.

$$\frac{d([C0] \cdot V_{ext})}{dt} = +V_{ext} \cdot (k2 \cdot [B1] \cdot 0.33 \cdot CA)$$
$$-(A \cdot Pc \cdot ([C0] - [C0c]))$$
$$+V_{ext} \cdot (k2 \cdot [B0] \cdot 1 \cdot CA)$$
$$-V_{ext} \cdot (k1 \cdot [C0] \cdot 1 \cdot CA)$$

$$\frac{d([C1] \cdot V_{ext})}{dt} = -V_{ext} \cdot (k1 \cdot [C1] \cdot 1 \cdot CA)$$
$$+V_{ext} \cdot (k2 \cdot [B1] \cdot 0.67 \cdot CA)$$
$$-(A \cdot Pc \cdot ([C1] - [C1c]))$$

$$\frac{d([B0] \cdot V_{ext})}{dt} = -(A \cdot Pb \cdot ([B0] - [B0c]))$$
$$-V_{ext} \cdot (k2 \cdot [B0] \cdot 1 \cdot CA)$$
$$+V_{ext} \cdot (k1 \cdot [C0] \cdot 1 \cdot CA)$$

$$\frac{d([B1] \cdot V_{ext})}{dt} = +V_{ext} \cdot (k1 \cdot [C1] \cdot 1 \cdot CA)$$
$$-V_{ext} \cdot (k2 \cdot [B1] \cdot 0.33 \cdot CA)$$
$$-V_{ext} \cdot (k2 \cdot [B1] \cdot 0.67 \cdot CA)$$
$$-(A \cdot Pb \cdot ([B1] - [B1c]))$$

$$\frac{d([C0c] \cdot V_{chl})}{dt} = +V_{chl} \cdot (k2 \cdot [B1c] \cdot 0.33 \cdot CAc)$$
$$+V_{chl} \cdot (k2 \cdot [B0c] \cdot 1 \cdot CAc)$$
$$+(A \cdot Pc \cdot ([C0] - [C0c]))$$
$$-V_{chl} \cdot (k1 \cdot [C0c] \cdot 1 \cdot CAc)$$

$$\frac{d([B1c] \cdot V_{chl})}{dt} = -V_{chl} \cdot (k2 \cdot [B1c] \cdot 0.33 \cdot CAc)$$
$$+(A \cdot Pb \cdot ([B1] - [B1c]))$$
$$+V_{chl} \cdot (k1 \cdot [C1c] \cdot 1 \cdot CAc)$$
$$-V_{chl} \cdot (k2 \cdot [B1c] \cdot 0.67 \cdot CAc)$$

$$\frac{d([C1c] \cdot V_{chl})}{dt} = +(A \cdot Pc \cdot ([C1] - [C1c]))$$
$$-V_{chl} \cdot (k1 \cdot [C1c] \cdot 1 \cdot CAc)$$
$$+V_{chl} \cdot (k2 \cdot [B1c] \cdot 0.67 \cdot CAc)$$

$$\frac{d([B0c] \cdot V_{chl})}{dt} = -V_{chl} \cdot (k2 \cdot [B0c] \cdot 1 \cdot CAc)$$
$$+(A \cdot Pb \cdot ([B0] - [B0c]))$$
$$+V_{chl} \cdot (k1 \cdot [C0c] \cdot 1 \cdot CAc)$$

Figure S3

Differential equations for numerical modelling used for the generation of time courses for changes in singly labelled CO_2 species shown in Figure 5, for the curve fitting in Figure 6 and permeabilities value presented in Table 1 and Table 2. The graphical output shown in the Figure 5 was generated from the mathematical output tool of the COPASI biochemical simulator program (copasi.org), which is described in detail in the methods. The symbols and units used in the equations are described in detail in Table S1.

COPASI file of numerical modelling for lowly enriched ^{18}O bicarbonate is available online: http://dx.doi.org/10.5061/dryad.2r05d

Parameter	units	Initial value	Description
Vext	m^3	1	volume of external compartment
Vchl	m^3	6.5E-5	volume of chloroplast compartment
C0	mol m^{-3}	0	external $^{13}C^{16}O_2$ - 45
C1	mol m^{-3}	0	external $^{13}C^{18}O^{16}O$ - 47
B0	mol m^{-3}	0.964	external $H^{13}C^{16}O_3^-$
B1	mol m^{-3}	0.0357	external $H^{13}C^{18}O^{16}O^{16}O^-$
C0c	mol m^{-3}	0	chloroplastic $^{13}CO_2$
C1c	mol m^{-3}	0	chloroplastic $^{13}C^{18}O^{16}O$
B0c	mol m^{-3}	0	chloroplastic $H^{13}C^{16}O_3^-$
B1c	mol m^{-3}	0	chloroplastic $H^{13}C^{18}O^{16}O^{16}O^-$
k1	s^{-1}	0.0221	CO_2 hydration rate constant external compartment
k2	s^{-1}	0.00584	bicarb dehydration rate constant external compartment
CA	unitless	1	external CA catalysis factor
CAc	unitless	1.00E+07	chloroplast CA catalysis factor
A	m^2 m^{-3}	0	area of chloroplast envelope per m^3 assay (0 to 30 m^2)
Pc	m s^{-1}	0.001	permeability of chloroplast envelope to CO_2
Pb	m s^{-1}	1.00E-06	permeability of chloroplast envelope to HCO_3^-

Table S1
Parameters and their units and values used for the model equations used in Figures S3.

$$\frac{d([C1]\cdot V_{ext})}{dt} = -V_{ext}\cdot([C1]\cdot k1\cdot CA) + V_{ext}\cdot([B1]\cdot k2\cdot CA\cdot 0.67) + V_{ext}\cdot([B2]\cdot k2\cdot CA\cdot 0.67) - (Pc\cdot A\cdot([C1]-[C1c]))$$

$$\frac{d([C0]\cdot V_{ext})}{dt} = -V_{ext}\cdot([C0]\cdot k1\cdot CA) + V_{ext}\cdot([B0]\cdot k2\cdot CA\cdot 1) + V_{ext}\cdot([B1]\cdot k2\cdot CA\cdot 0.33) - (Pc\cdot A\cdot([C0]-[C0c]))$$

$$\frac{d([C2]\cdot V_{ext})}{dt} = -V_{ext}\cdot([C2]\cdot k1\cdot CA) + V_{ext}\cdot([B2]\cdot k2\cdot CA\cdot 0.33) + V_{ext}\cdot([B3]\cdot k2\cdot CA\cdot 1) - (Pc\cdot A\cdot([C2]-[C2c]))$$

$$\frac{d([B0]\cdot V_{ext})}{dt} = +V_{ext}\cdot([C0]\cdot k1\cdot CA) - V_{ext}\cdot([B0]\cdot k2\cdot CA\cdot 1) - (Pb\cdot A\cdot([B0]-[B0c]))$$

$$\frac{d([B1]\cdot V_{ext})}{dt} = +V_{ext}\cdot([C1]\cdot k1\cdot CA) - V_{ext}\cdot([B1]\cdot k2\cdot CA\cdot 0.33) - V_{ext}\cdot([B1]\cdot k2\cdot CA\cdot 0.67) - (Pb\cdot A\cdot([B1]-[B1c]))$$

$$\frac{d([B2]\cdot V_{ext})}{dt} = +V_{ext}\cdot([C2]\cdot k1\cdot CA) - V_{ext}\cdot([B2]\cdot k2\cdot CA\cdot 0.67) - V_{ext}\cdot([B2]\cdot k2\cdot CA\cdot 0.33) - (Pb\cdot A\cdot([B2]-[B2c]))$$

$$\frac{d([B3]\cdot V_{ext})}{dt} = -V_{ext}\cdot([B3]\cdot k2\cdot CA\cdot 1) - (Pb\cdot A\cdot([B3]-[B3c]))$$

$$\frac{d([B3c]\cdot V_{chl})}{dt} = -V_{chl}\cdot([B3c]\cdot k2\cdot CAc\cdot 1) + (Pb\cdot A\cdot([B3]-[B3c]))$$

$$\frac{d([B2c]\cdot V_{chl})}{dt} = +V_{chl}\cdot([C2c]\cdot k1\cdot CAc) - V_{chl}\cdot([B2c]\cdot k2\cdot CAc\cdot 0.67) - V_{chl}\cdot([B2c]\cdot k2\cdot CAc\cdot 0.33) + (Pb\cdot A\cdot([B2]-[B2c]))$$

$$\frac{d([B1c]\cdot V_{chl})}{dt} = +V_{chl}\cdot([C1c]\cdot k1\cdot CAc) - V_{chl}\cdot([B1c]\cdot k2\cdot CAc\cdot 0.33) - V_{chl}\cdot([B1c]\cdot k2\cdot CAc\cdot 0.67) + (Pb\cdot A\cdot([B1]-[B1c]))$$

$$\frac{d([B0c]\cdot V_{chl})}{dt} = +V_{chl}\cdot([C0c]\cdot k1\cdot CAc) - V_{chl}\cdot([B0c]\cdot k2\cdot CAc\cdot 1) + (Pb\cdot A\cdot([B0]-[B0c]))$$

$$\frac{d([C2c]\cdot V_{chl})}{dt} = -V_{chl}\cdot([C2c]\cdot k1\cdot CAc) + V_{chl}\cdot([B2c]\cdot k2\cdot CAc\cdot 0.33) + V_{chl}\cdot([B3c]\cdot k2\cdot CAc\cdot 1) + (Pc\cdot A\cdot([C2]-[C2c]))$$

$$\frac{d([C1c]\cdot V_{chl})}{dt} = -V_{chl}\cdot([C1c]\cdot k1\cdot CAc) + V_{chl}\cdot([B1c]\cdot k2\cdot CAc\cdot 0.67) + V_{chl}\cdot([B2c]\cdot k2\cdot CAc\cdot 0.67) + (Pc\cdot A\cdot([C1]-[C1c]))$$

$$\frac{d([C0c]\cdot V_{chl})}{dt} = -V_{chl}\cdot([C0c]\cdot k1\cdot CAc) + V_{chl}\cdot([B0c]\cdot k2\cdot CAc\cdot 1) + V_{chl}\cdot([B1c]\cdot k2\cdot CAc\cdot 0.33) + (Pc\cdot A\cdot([C0]-[C0c]))$$

Figure S4

Differential equations for numerical modelling used for the generation of time courses for changes in double labelled CO_2 species shown in Figure S2. The graphical output shown in the Figure S2 was generated from the mathematical output tool of the COPASI biochemical simulator program (copasi.org), which is described in detail in the methods. The symbols and units used in the equations are described in detail in Table S2.

COPASI file of numerical modelling for highly enriched ^{18}O bicarbonate is available online: http://dx.doi.org/10.5061/dryad.2r05d

Parameter	units	Initial value	Description
Vext	m^3	1	volume of external compartment
Vchl	m^3	6.5E-05	volume of chloroplast compartment
C0	mol m^{-3}	0	external $^{13}C^{16}O_2$ - 45
C1	mol m^{-3}	0	external $^{13}C^{18}O^{16}O$ - 47
C2	mol m^{-3}	0	external $^{13}C^{18}O^{18}O$ - 49
B0	mol m^{-3}	0	external $H^{13}C^{16}O_3^-$
B1	mol m^{-3}	0	external $H^{13}C^{18}O^{16}O^{16}O^-$
B2	mol m^{-3}	0	external $H^{13}C^{18}O^{18}O^{16}O^-$
B3	mol m^{-3}	1	external $H^{13}C^{18}O^{18}O^{18}O^-$
C0c	mol m^{-3}	0	chloroplastic $^{13}CO_2$
C1c	mol m^{-3}	0	chloroplastic $^{13}C^{18}O^{16}O$
C2c	mol m^{-3}	0	chloroplastic $^{13}C^{18}O^{18}O$ - 49
B0c	mol m^{-3}	0	chloroplastic $H^{13}C^{16}O_3^-$
B1c	mol m^{-3}	0	chloroplastic $H^{13}C^{18}O^{16}O^{16}O^-$
B2c	mol m^{-3}	0	chloroplastic $H^{13}C^{18}O^{18}O^{16}O^-$
B3c	mol m^{-3}	0	chloroplastic $H^{13}C^{18}O^{18}O^{18}O^-$
k1	s^{-1}	0.05	CO_2 hydration rate constant external compartment
k2	s^{-1}	0.001	bicarb dehydration rate constant external compartment
CA	unitless	1	external CA catalysis factor
CAc	unitless	1.00E+07	chloroplast CA catalysis factor
A	m^2 m^{-3}	30	area of chloroplast envelope per m^3 assay (0 or 30)
Pc	m s^{-1}	0.001	permeability of chloroplast envelope to CO_2
Pb	m s^{-1}	1.00E-06	permeability of chloroplast envelope to HCO_3^-

Table S2
Parameters and their units and values used for the model equations used in Figures S4.

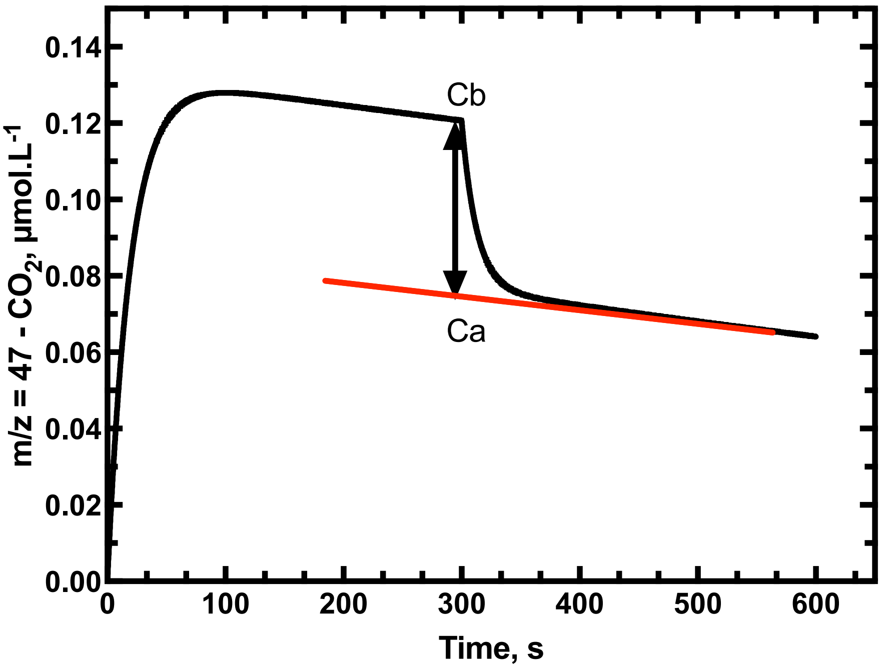

Figure S5
Estimation of P_{CO_2} from chloroplast injection time courses (injection at 300s). Data in the figure were generated by modelling and the procedure requires two points to be estimated, Cb (CO_2 concentration immediately before chloroplast injection; and Ca, the extrapolation of the CO_2 time course after injection back to the injection time point.

P_{CO_2} =(Cb-Ca).k_h/(A.Ca)

Where Pc is CO_2 permeability, m s^{-1}; Cb, initial [CO_2], µmol L^{-1}; Ca, [CO_2] after, µmol L^{-1}; k_h, CO_2 hydration rate constant, s^{-1}; k_d, HCO_3^- dehydration rate constant, s^{-1}; A, envelope area of injected chloroplasts, $m^2.m^{-3}$, which was 30 in this case.

Derivation of the above equation is given below.
Immediately before chloroplast injection it is assumed that:

$$\frac{d[^{13}C^{18}O^{16}O]}{dt} = [H^{13}C^{18}O^{16}O^{16}O^-].\frac{2}{3}\,k_d - [^{13}C^{18}O^{16}O].k_h \qquad (1)$$

After injection of chloroplasts when steady state exchange is reached (after 400 s)

$$\frac{d[^{13}C^{18}O^{16}O]}{dt} = [H^{13}C^{18}O^{16}O^{16}O^-].\frac{2}{3}\,k_d - [^{13}C^{18}O^{16}O].k_h - A.\,P_{CO_2}\,.\,[^{13}C^{18}O^{16}O] (2)$$

if steady state $\frac{d[^{13}C^{18}O^{16}O]}{dt}$ is assumed to be approximately the same before and after addition of chloroplasts (this assumes the HCO_3^- permeability is very low and HCO_3^- species are equal before and after injection) and CO_2 extrapolated back to zero is estimated as shown in the above figure (Ca) then it can be assumed that equation 1 = equation 2 and rearrangement yields:

$$Cb.k_h = Ca.k_h + A.\,P_{CO_2}\,.Ca$$

And thus

$$P_{CO_2} = \frac{(Cb-Ca)}{Ca}.\frac{k_h}{A} \qquad (3)$$

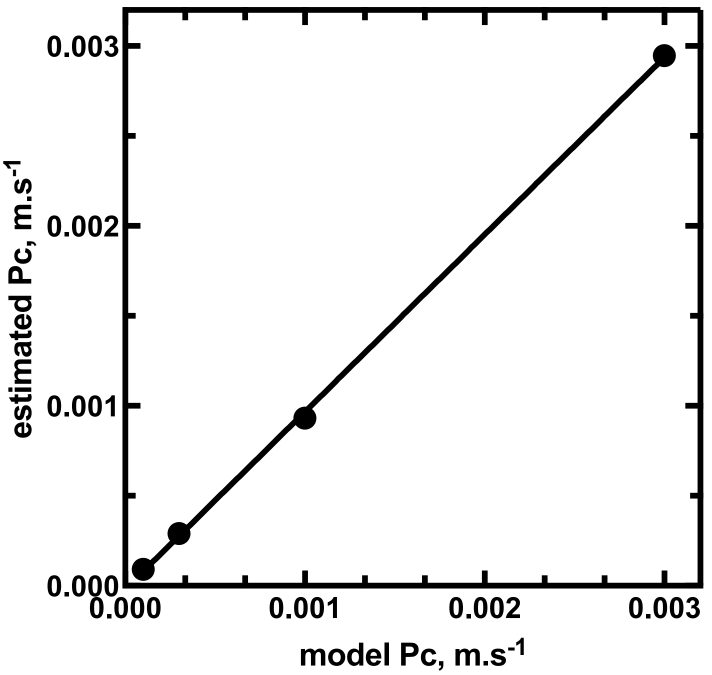

Figure S6

Empirical verification of the graphical estimation procedure for the method and equation shown in Figure S3. Model data was generated for four different P_{CO_2} values shown in the figure. The area of chloroplasts envelope per m^3 assay injected was 30 m^2 m^{-3} and $P_{HCO_3^-}$ was set at 10^{-8} m s^{-1}. Cb, Ca and P_{CO_2} values were estimated graphically as described in Figure S5 and the data used was from Figure 5. The estimated P_{CO_2} is plotted against the model value for P_{CO_2} and a straight line fit is drawn.

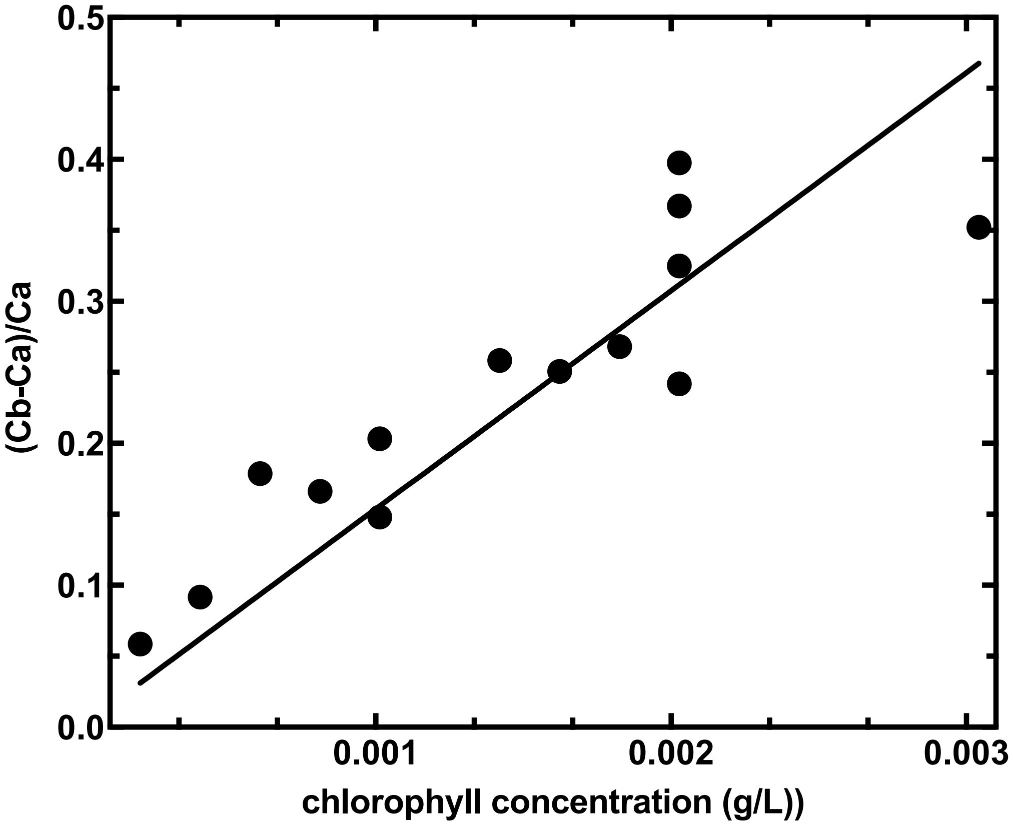

Figure S7
Correlation between chloroplast number (chlorophyll concentration) and determination of permeability values before correction by chloroplast number. Data have been accumulated over five independent experiments.

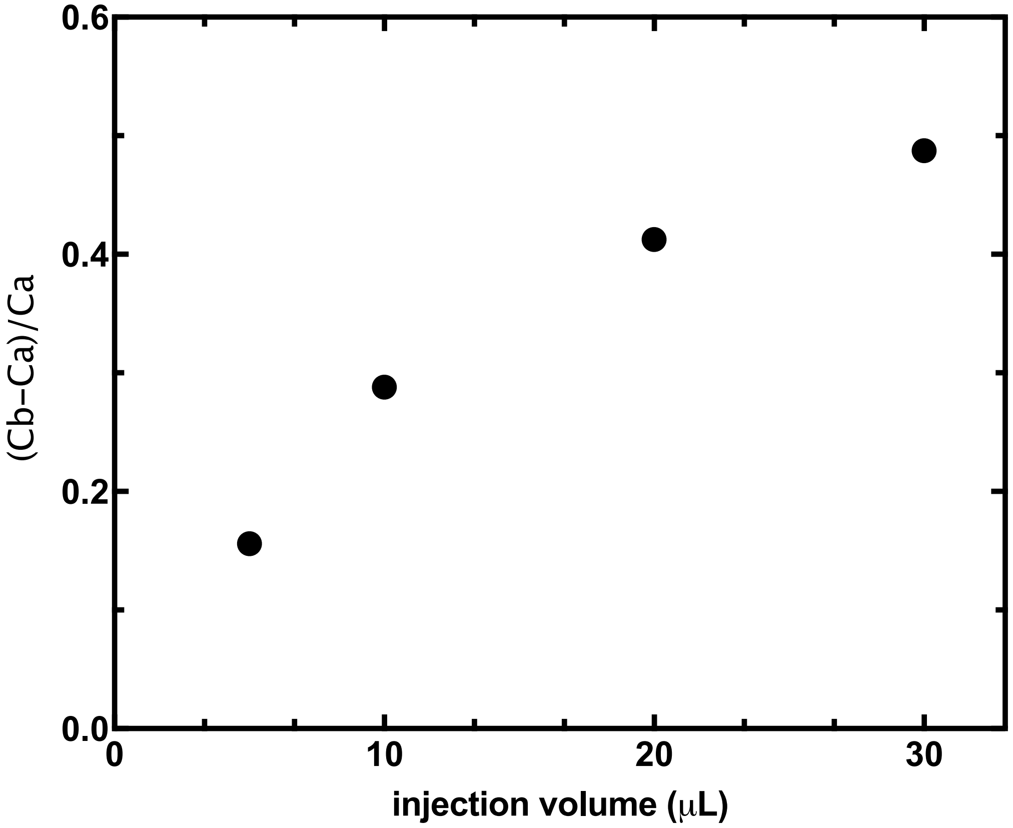

Figure S8
Correlation between volume of chloroplasts injected in the MIMS cuvette and the drop of $^{13}C^{18}O^{16}O$ (m/z = 47) at the injection, as calculated in Fig. S5.

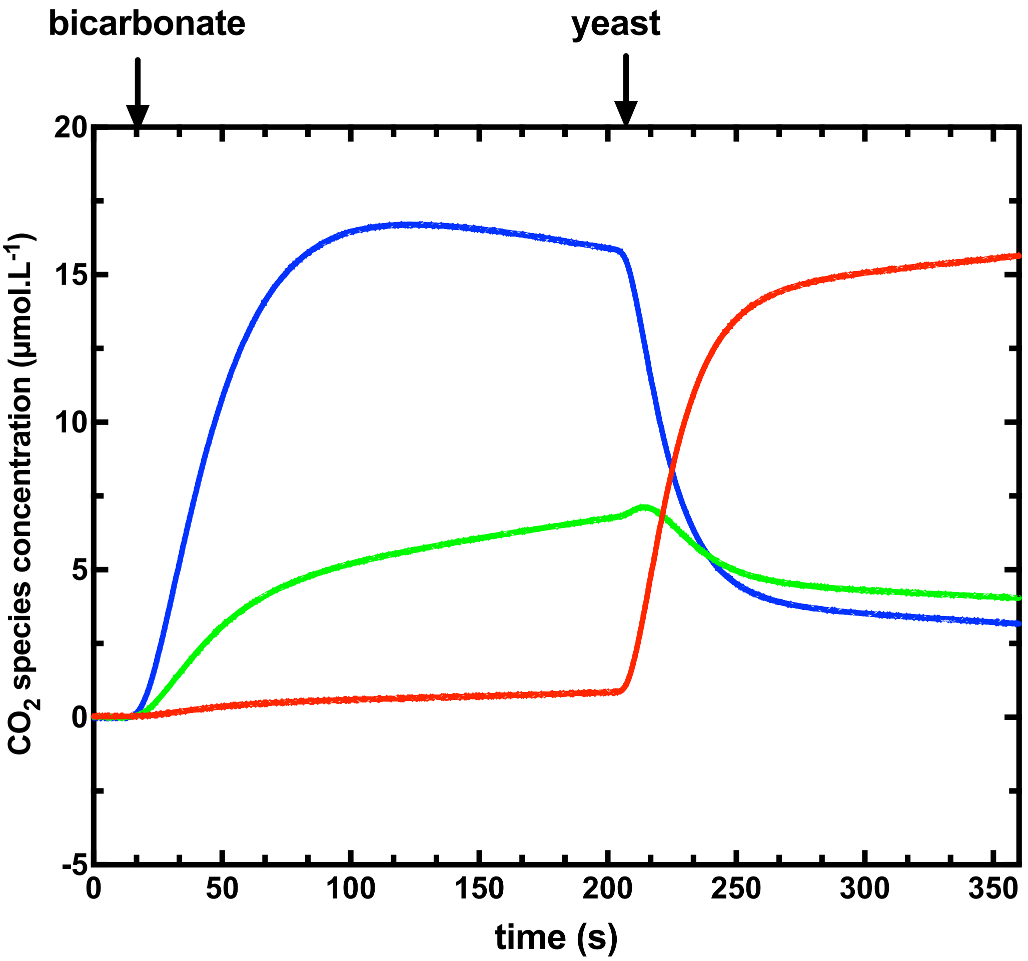

Figure S9
Typical time course for a highly ^{18}O enriched assay. Changes in concentrations of $^{13}C^{18}O^{18}O$ (m/z = 49, blue), $^{13}C^{18}O^{16}O$(m/z = 47, green), $^{13}C^{16}O^{16}O$ (m/z = 45, red) species are shown. After injection of highly ^{18}O-enriched Ci (equilibrated against 1% ^{18}O water), chemical equilibrium is reached before yeast injection at 210 s.
